# Predatory publishers: Time for action

**DOI:** 10.1002/nop2.104

**Published:** 2017-10-05

**Authors:** Roger Watson

Plenty is being written about predatory publishers (Clark & Thompson, 2017; Pickler et al., 2015) but little is being done to stop them. The one valiant effort to expose them: Beall's List of Predatory publishers, has been forcibly removed from the Internet (Watson, 2017a), and while someone else has made the list available online (http://beallslist.weebly.com/; accessed 18 September 2017), this list is static. Cabell's International (http://www.cabells.com/; accessed 18 September 2017) does produce a list of predatory journals but, unlike Beall's List, this is not available free. Darbyshire, McKenna, Lee, and East (2017) issued a call to take a stand against predatory publishers but there seems to be little action by institutions to lever an end to the predatory publishing “industry” and this was the focus of my recent article (Watson, 2017b) in the *Times Higher Education*. One letter to the *Times Higher Education* the following week was not supportive, citing the profits of the academic publishing industry and that university libraries and information services do provide advice. This seemed to miss the point of my article and I am using the editorial pages of *Nursing Open* to reiterate my view.

Predatory publishers harm academics, are not good for academic publishing and they publish poor quality work which, generally and demonstrably, has not been peer reviewed. They make false claims about their impact factors (Jalalian, 2015) and editorial board memberships (Sorokowski, Kulczycki, Sorokowska, & Pisnaki, 2017) thereby misleading some unwary academics to publish with them. This activity—purporting to provide a service which they do not provide—can accurately be called fraud. My view is that universities ought to be getting much tougher regarding policies around publishing generally, and specifically, they should take some action to prevent academics from publishing in predatory journals and censure academics who do. Towards that end I suggest a list of “action points”:

1. Universities should provide training and updates for all academic staff on predatory publishing
2. Universities should develop policies regarding predatory publishing
3. Universities should produce lists of approved journals for their subject groups
4. Universities should discount articles published in predatory journals from CVs for appointments and promotions

Drawing up lists of approved journals would not be hard. Many lists of journals exist where their quality in terms of peer review and ethical practices have been evaluated. Such lists include: Thomson Reuters list of journals assigned an impact factor and also their Emerging Sources Citation Index; PubMed; Directory of Open Access Journals; and Web of Science. However, these are only a few examples in a limited number of fields. There would hardly be a case for drastic disciplinary action against staff unless they persistently “offended” despite best efforts to advise them. If universities—and other bodies such as research funders—took sufficient action, then the predatory publishers would be starved of funds and they would cease to exist.
